# Pharmacological Basis for Use of *Armillaria mellea* Polysaccharides in Alzheimer's Disease: Antiapoptosis and Antioxidation

**DOI:** 10.1155/2017/4184562

**Published:** 2017-09-10

**Authors:** Shengshu An, Wenqian Lu, Yongfeng Zhang, Qingxia Yuan, Di Wang

**Affiliations:** ^1^School of Life Sciences, Jilin University, Changchun 130012, China; ^2^Zhuhai College, Jilin University, Zhuhai 519041, China

## Abstract

*Armillaria mellea*, an edible fungus, exhibits various pharmacological activities, including antioxidant and antiapoptotic properties. However, the effects of *A. mellea* on Alzheimer's disease (AD) have not been systemically reported. The present study aimed to explore the protective effects of mycelium polysaccharides (AMPS) obtained from *A. mellea*, especially AMPSc via 70% ethanol precipitation in a L-glutamic acid- (L-Glu-) induced HT22 cell apoptosis model and an AlCl_3_ plus D-galactose- (D-gal-) induced AD mouse model. AMPSc significantly enhanced cell viability, suppressed nuclear apoptosis, inhibited intracellular reactive oxygen species accumulation, prevented caspase-3 activation, and restored mitochondrial membrane potential (MMP). In AD mice, AMPSc enhanced horizontal movements in an autonomic activity test, improved endurance times in a rotarod test, and decreased escape latency time in a water maze test. Furthermore, AMPSc reduced the apoptosis rate, amyloid beta (A*β*) deposition, oxidative damage, and p-Tau aggregations in the AD mouse hippocampus. The central cholinergic system functions in AD mice improved after a 4-week course of AMPSc administration, as indicated by enhanced acetylcholine (Ach) and choline acetyltransferase (ChAT) concentrations, and reduced acetylcholine esterase (AchE) levels in serum and hypothalamus. Our findings provide experimental evidence suggesting *A. mellea* as a neuroprotective candidate for treating or preventing neurodegenerative diseases.

## 1. Introduction

Devastating neurodegenerative disorders, such as Alzheimer's disease (AD), are caused by neuronal loss and synapse degeneration. These disorders are clinically characterized by learning and memory decline, as well as cognitive deficits, and no cure is currently available [1]. The neuronal losses observed in neurodegenerative diseases are attributable to the oxidative death of these oxidative stress-sensitive cells [2]. Oxidative stress promotes neurotoxicity by increasing amyloid beta (A*β*) aggregation concomitantly with inflammatory events such as reactive oxygen species (ROS) production [3]. Additionally, excess extracellular glutamate levels have been found to correlate with the development of neurodegenerative disorders by triggering oxidative glutamate damage, preventing the intracellular antioxidant synthesis, and ultimately leading to ROS accumulation [4]. The overproduction of ROS and A*β* causes a feedback loop that results in synaptic dysfunction, as well as mitochondria-mediated apoptosis [5]. Therefore, antioxidant therapies are being considered as new options for protecting neurons from the oxidative damage associated with AD. These antioxidants not only can scavenge free radicals but may also reduce damage due to oxidative stress and thus maintain the cellular redox balance [6].

Several types of fungus are currently used as functional foods. In addition, many exhibit pharmacological activities with few side effects and are used as medicinal agents. Encouragingly, many fungal species have been reported to display neuroprotective properties in the context of neurodegenerative diseases [7]. Our group found that a polysaccharide isolated from *Sparassis crispa* protected PC12 cells against L-glutamic acid- (L-Glu-) induced apoptosis via the mitochondrial apoptotic pathway [8]. Furthermore, aqueous extracts of *Hericium erinaceus* yielded therapeutic effects that were attributed to both mitochondria-mediated apoptosis and neurotransmitter modulation in apoptotic cells and in an AlCl_3_ plus D-galactose- (D-gal-) induced mouse model of AD [9]. *Armillaria mellea*, an edible and medicinal fungus, has been used for hundreds of years in East Asia. Polysaccharides isolated from *A. mellea* have been reported to exhibit antioxidant activities by superoxide radical scavenging [10] and significant antitumor activities via the mitochondrial apoptotic pathway and caspase cascade activation [11]. All previous data have indicated that *A. mellea* may exert protective effects against neurodegenerative diseases, especially AD.

The neurotoxin-induced mouse hippocampal neuronal cell (i.e., HT22 cell) apoptosis model is a well-recognized *in vitro* model for screening the neuroprotective effects of various agents [12]. Additionally, an aging model induced by D-gal is used in animal studies. This model involves the blocking of natural physiological features of aging and exhibits cellular AD phenomena, including a wide range of astrocytic and neuronal vacuolization, neuronal degeneration or death, and A*β* production and deposition, followed by cerebral cortex atrophy and cognitive and memory dysfunction [13]. The use of a combination of AlCl_3_ and D-gal in a mouse model induces AD-like behavior and more readily generates pathological alterations than either AlCl_3_- or D-gal-only treatment [14].

In the present study, we used L-Glu-induced HT22 apoptotic cells and D-gal plus AlCl_3_-induced AD mice to investigate the neuroprotective effects of *A. mellea* mycelium polysaccharides (AMPS). We found that in HT22 cells, AMPS improved cell viability, restored mitochondrial membrane potential (MMP), and reduced cell apoptosis and excess caspase-3 activity. Moreover, AMPS treatment regulated the behavior and physiological and biochemical indexes of AD mice. Taken together, our data suggest the usefulness of *A. mellea* as a therapeutic agent or functional food for the treatment of AD.

## 2. Materials and Methods

### 2.1. Preparation of *A. mellea* Polysaccharides

**Figure 1:**
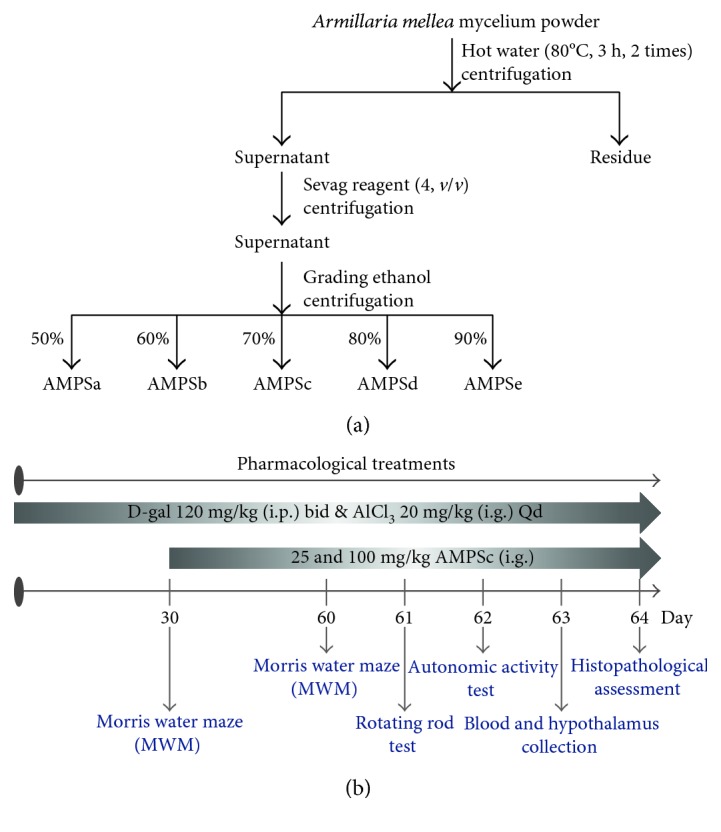
(a) The preparation of polysaccharides isolated from *A. mellea* mycelium obtained via submerged fermentation. (b) The process of AlCl_3_ combined with D-gal-induced Alzheimer's disease mouse model establishment and drug administration.

*A. mellea* (CICC 14066; China Center of Industrial Culture Collection, Beijing, China) mycelium was obtained through submerged fermentation with the medium consisted of 20 g/L of sucrose, 10 g/L of glucose, 10 g/L yeast extract powder, 10 g/L of peptone, 1.5 g/L of KH_2_PO_4_, 0.75 g/L of MgSO_4_, and 0.01 g/L of vitamin B1. *A. mellea* was extracted by hot water at 80°C for 3 h twice, removed proteins using Sevag reagent (n-butanol and chloroform in 1 : 4 ratio), and then collected after precipitation using 50%, 60%, 70%, 80%, and 90% ethanol at 4°C overnight and named AMPSa–e (Figure 1(a)). The yield of polysaccharides within *A. mellea* mycelium was shown in Table 1.

### 2.2. Cell Culture

The mouse hippocampal neuronal cell line (HT22; BNCC; 337709) was cultured in Dulbecco's modified Eagle's medium (DMEM; Invitrogen, USA) supplemented with 10% fetal bovine serum (FBS, Invitrogen, USA), 100 *μ*g/mL streptomycin, and 100 units/mL penicillin (Invitrogen, USA) in a 5% CO2 and 95% air incubator supplying a humidified atmosphere at 37°C. Before treatment, HT22 cells were differentiated in Neurobasal medium (Invitrogen) containing 2 mmol/L glutamine and 1× N_2_ supplement (Invitrogen) for 24 hours [15].

### 2.3. Cell Viability Assay

HT22 cells were pretreated with AMPSa–e at a dose of 40 *μ*g/mL or AMPSc at doses of 10, 20, 40, and 80 *μ*g/mL for 3 h and then incubated with 25 mM of L-GLu for 24 h. 3-(4,5-Dimethyl-2-thiazolyl)-2,5-diphenyl-2H-tetrazolium bromide assay (MTT, Sigma-Aldrich, USA) was applied for cell viability assessment similarly as previous research [8].

### 2.4. Cell Apoptosis Assay

HT22 cells were pretreated with AMPSc at doses of 40 and 80 *μ*g/mL for 3 h and then incubated with 25 mM of L-Glu for another 24 h. Cells were then incubated with propidium iodide (PI) and annexin V (AV) for 20 min at room temperature in darkness. The intensity of fluorescence was measured utilizing Muse™ Cell Analyzer from Millipore (Billerica, MA) following manufacturer's instructions.

### 2.5. MMP Assay

Cells were pretreated with AMPSc (40 and 80 *μ*g/mL) for 3 h and then exposed to 25 mM of L-Glu for another 12 h and then incubated with JC-1 (5,5′,6,6′-tetrachloro-1,1′,3,3′tetraethylbenzimidazol-ylcarbocyanine iodide) at 37°C for 20 min in darkness. The ratio of green/red fluorescence analyzed using Muse Cell Analyzer (Millipore; USA) indicated the value of mitochondrial membrane potential.

### 2.6. Intracellular ROS Generation Assessment

HT22 cells were pretreated with AMPSc (40 and 80 *μ*g/mL) for 3 h and then exposed to 25 mM of L-Glu for another 12 h. Treated cells were incubated with 10 *μ*mol/L of 2′,7′-dichlorofluorescein diacetate (DCFH-DA) at 37°C for 20 minutes. Green fluorescence intensity detected with a fluorescent microscope (40x; CCD camera, IX73, Olympus) represented the level of intracellular ROS.

### 2.7. Assessment of Caspase Activities

HT22 cells were pretreated with AMPSc (40 and 80 *μ*g/mL) for 3 h and then exposed to 25 mM of L-Glu for another 24 h. The activities of caspase-3 were analyzed via commercial kits (Nanjing Jiancheng Bioengineering Institute, Nanjing, China).

### 2.8. Experiments Applied on Alzheimer's Disease Mouse Model

The experiments were carried out under the approval of Institution Animal Ethics Committee of Jilin University. 50 Balb/c male mice (20–22 g; 10 weeks) were housed in cages in an air-conditioned room under temperature (23 ± 1°C) and humidity (40–60%) with sufficient water and food and randomly divided into five groups (*n* = 10). 30 mice were subcutaneously injected with 120 mg/kg of D-gal and orally treated with 20 mg/kg of AlCl_3_ once a day for 8 weeks. Starting from the fifth week, mice were intragastrically treated with normal saline (model group) and AMPSc at doses of 25 and 100 mg/kg/day for four weeks. 10 mice serving as control group were treated with normal saline for 8 weeks. Another 10 mice were treated with normal saline for 4 weeks, following with 100 mg/kg of AMPSc administration for another 4 weeks (Figure 1(b)).

At the end of behavioral tests as follows, blood was collected from the rats' tails under anesthesia with 10% chloral hydrate, and the brains were removed and homogenized (1 : 9 *w*/*v*) in NaCl buffer. The whole hemisphere was immersed in 4% formaldehyde for pathologic analysis.

### 2.9. Behavioral Tests

#### 2.9.1. Morris Water Maze Test

Memory ability and spatial learning were analyzed by Morris water maze (MT-200, Chengdu, China). After 5-day training, on the 60th day, mice were put into a circular pool filled with opacified water containing titanium dioxide (23 ± 2°C, 10 cm in depth). The escape latency of mice to find the platform was recorded within 120 s.

#### 2.9.2. Fatigue Rotarod Test

On the 61st day, after 3 times training, mice were placed on the turning device (ZB-200, Chengdu Techman Software Co. Ltd., Chengdu, China) with 15 rpm speed, and the time when mice under induced muscle fatigue fell off was recorded.

#### 2.9.3. Autonomic Activity Test

On the 62nd day, mice were placed in the chamber covered with the light-blocking plate to detect their autonomic activities. The number of mouse activities including the horizontal movements and the vertical movements was recorded for 5 min.

### 2.10. Determination of the Levels of Ach, AchE, and ChAT in Serum and Hypothalamus

The levels of acetylcholine (Ach), acetylcholine esterase (AchE), and choline acetyltransferase (ChAT) in serum and hypothalamus were measured by enzyme-linked immunosorbent assay (ELISA) according to the procedures provided by the related assay kits (Nanjing Jiancheng Bioengineering Institute, Nanjing, China).

### 2.11. Determination of Oxidation Status in Serum or Hypothalamus

The levels of superoxide dismutase (SOD), glutathione peroxidase (GSH-Px), and ROS in serum and/or hypothalamus were detected by ELISA kit according to related procedures (Nanjing Jiancheng Bioengineering Institute, Nanjing, China).

### 2.12. TUNEL Assay

Apoptosis in the hippocampus was detected using the terminal deoxynucleotidyl transferase-mediated dUTP nick end labeling (TUNEL). After deparaffinization, hippocampus tissue sections were washed twice in phosphate-buffered saline (PBS) for 5 minutes and completely covered by the permeabilization reagent (Proteinase K) for 15 min at room temperature. After washing with PBS, sections were incubated with 50 *μ*L of the prepared TdT reaction mixture at 37°C for 60 min in the dark. The reactions were subsequently terminated, and the tissue sections were analyzed under a Nikon Eclipse TE 2000-S fluorescence microscope (20x; CCD camera, IX73, Olympus).

### 2.13. Determination of Levels of A*β* in Serum and Hippocampus

The levels of A*β*1-42 in serum were detected by ELISA kit according to related procedures (Nanjing Jiancheng Bioengineering Institute, Nanjing, China).

Brain coronal sections were deparaffinized, placed in thioflavin-S solution for 5 min, and then differentiated in 70% fresh alcohol for 10 min. After washing, images were captured using a confocal microscope (40x; CCD camera, IX73, Olympus).

### 2.14. Immunohistochemistry

The protein expressions of A*β*1-40, phospho- (p-) Tau (ser404), and 4-hydroxynonenal (4-HNE) in the hippocampus of mice were detected via immunohistochemistry to visualize A*β* deposition, tau aggregations, and oxidative stress-associated damage. The paraffin sections were deparaffinized in xylene and rehydrated in different graded alcohol. Then, sections were heated in antigen repair solution (citrate buffer) in a microwave for 20 min to retrieve antigens. After extensive washing with PBS for 5 min, sections were incubated with 3% hydrogen peroxide for 10 min at room temperature to block endogenous peroxidase followed by blocking with 2% goat serum dissolved in PBS. The slides were incubated with polyclonal anti-A*β*1-40 (1 : 200, Bioss bs-0877R); anti-p-Tau (ser404) (1 : 200, Bioss bs-2392R); and anti-4-HNE (1 : 800, Abcam: ab46545) antibodies individually overnight at 4°C. Subsequently, slides were washed with PBS and incubated with secondary antibody conjugated horseradish peroxidase (HRP) at room temperature for 1 h. And then, the sections were washed in PBS and visualized with DAB (3,3′-diaminobenzidine) (Solarbio, Beijing, China) followed by incubating with Mayer's hematoxylin for 3 min. Finally, the sections were dehydrated with dilutions of ethanol and xylene and digitized using an Olympus IX73 microscope (Olympus, Tokyo, Japan).

### 2.15. Statistical Analysis

Data were expressed as mean ± S.E.M. A one-way analysis of variance (ANOVA) was used to detect statistical significance followed by post hoc multiple comparisons (Dunn's test). Statistical significance was accepted for *P* < 0.05.

## 3. Results

### 3.1. AMPS Improved Cell Viability and Apoptosis and Reduced Caspase-3 Activity

**Figure 2:**
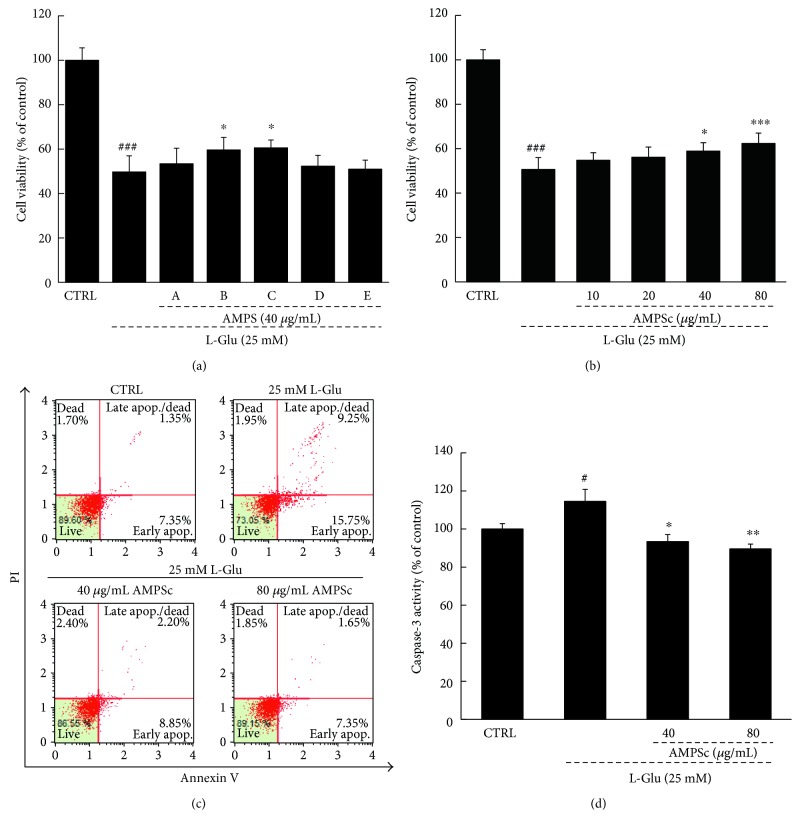
The neuroprotective effects of AMPS against L-Glu-induced cell damage in HT22 cells. (a) 3 h AMPSb and c preincubation improved cell viability in L-Glu-exposed HT22 cells. (b) 3 h AMPSc (40 and 80 μg/mL) pretreatment improved cell viability in HT22 cells after 24 h incubation with 25 mM of L-Glu. (c) 3 h AMPSc preincubation strongly reduced the apoptotic rate of HT22 cells exposed to L-Glu for 24 h. (d) 3 h AMPSc pretreatment weakened caspase-3 activations in HT22 cells exposed to 25 mM of L-Glu for 24 h. Data were expressed as a percentage of corresponding control cells and means ± S.E.M. (*n* = 6). ^#^*P* < 0.05 and ^###^*P* < 0.001 versus CTRL; ^∗^*P* < 0.05, ^∗∗^*P* < 0.01, and ^∗∗∗^*P* < 0.001 versus L-Glu-exposed cells. AMPS: *A. mellea* polysaccharides.

Compared with L-Glu-treated cells, cells pretreated with 40 *μ*g/mL of AMPSb and AMPSc for 3 h improved HT22 cell viability by 10% and 11%, respectively (*P* < 0.05; Figure 2(a)), whereas treatment with AMPSa, d, and e had no effect. Additionally, pretreatment with 40 or 80 *μ*g/mL of AMPSc for 3 h before a 24 h incubation with 25 mM of L-Glu improved HT22 cell viability by 6.9% and 13.7%, respectively (*P* < 0.05; Figure 2(b)). AV-PI staining revealed that whereas exposure to 25 mM L-Glu led to an apoptosis rate of 25% in HT22 cells, a 3 h preincubation with AMPSc led to a reduction in apoptosis of >14% (Figure 2(c)). When compared to L-Glu-damaged HT22 cells, a 3 h AMPSc pretreatment reduced caspase-3 activity by >24% during a 24 h incubation (Figure 2(d)).

### 3.2. AMPSc Restored the Dissipation of MMP and Reduced ROS Accumulation

**Figure 3:**
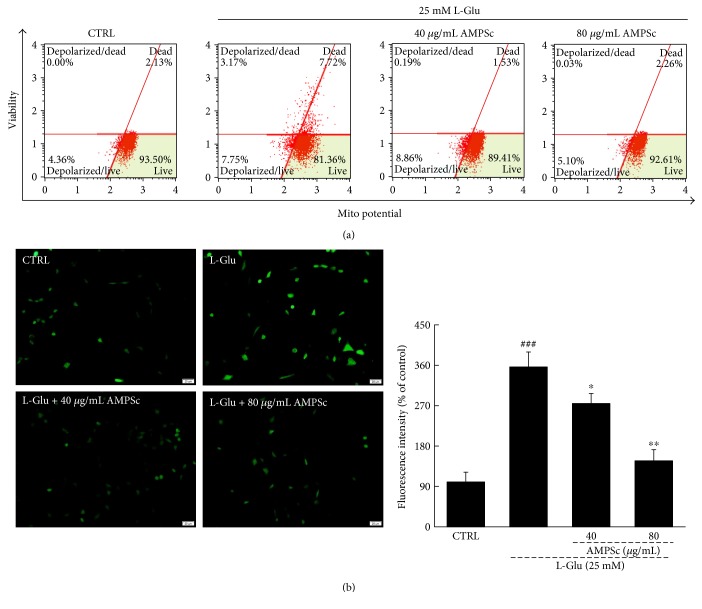
(a) The dissipation of MMP caused by 12 h L-Glu incubation was strongly restored by 3 h AMPSc pretreatment analyzing via JC-1 staining (*n* = 6). (b) The overproduction of ROS induced by 12 h L-Glu exposure was significantly decreased by 3 h AMPSc pretreatment analyzed by DCFH-DA staining (*n* = 6). Scale bar: 20 *μ*m. Qualification data were expressed as the percentage of green fluorescent intensity compared to control cells. Data are expressed as means ± S.E.M. (*n* = 6). ^###^*P* < 0.001 versus CTRL; ^∗^*P* < 0.05 and ^∗∗^*P* < 0.01 versus L-Glu-exposed cells. AMPS: *A. mellea* polysaccharides.

Altered mitochondrial apoptosis, which is characterized by disruption of the MMP, is a common feature of cell apoptosis [8]. Compared with L-Glu-damaged HT22 cells, AMPSc improved MMP depolarization by nearly 10% after a 12 h incubation (Figure 3(a)). Furthermore, a 3-h AMPSc pretreatment strongly suppressed L-Glu-induced ROS accumulation, as indicated by reduced green fluorescence (Figure 3(b)).

### 3.3. AMPSc Affected the Behavior of AD Mice

**Figure 4:**
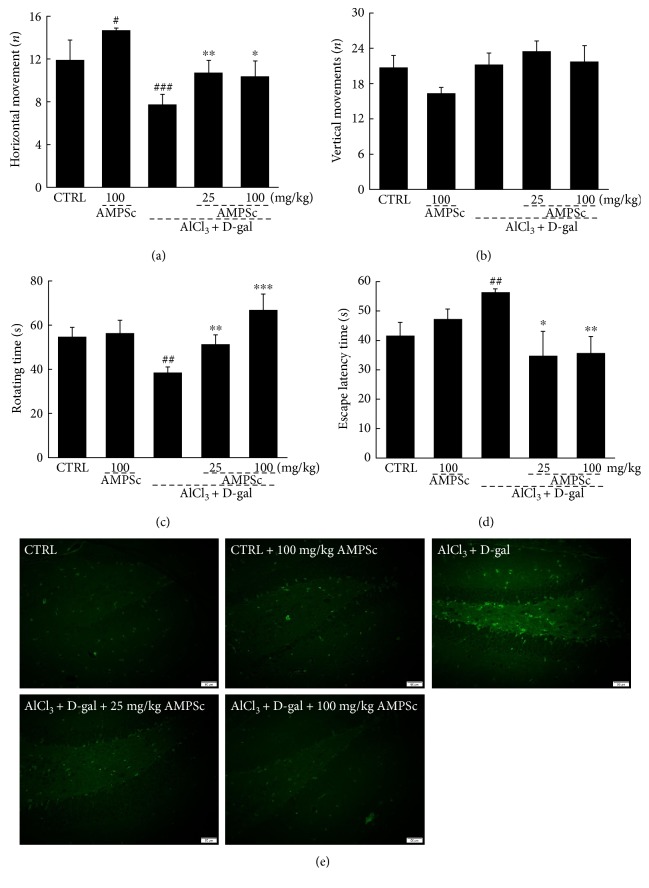
AMPSc improved AD-like behaviors in AlCl_3_ and D-gal induced AD mice. AMPSc enhanced (a) horizontal movements, but not (b) vertical movements in autonomous activity test, (c) prolonged endurance time in rotarod test, and (d) decreased escape latency time in water maze test in AD mice. Data are expressed as mean ± S.E.M. (*n* = 10). ^#^*P* < 0.05, ^##^*P* < 0.01, and ^###^*P* < 0.001 versus normal mice (CTRL); ^∗^*P* < 0.05, ^∗∗^*P* < 0.01, and ^∗∗∗^*P* < 0.001 versus AD mice. (e) AMPSc reduced apoptotic cell rate in the hippocampus of AD mice determined by TUNEL assay (*n* = 6). Scale bar: 50 *μ*m. AMPS: *A. mellea* polysaccharides.

We next subjected D-gal plus AlCl_3_-induced AD model mice for behavioral testing to further confirm the beneficial activities of AMPSc against AD. In an autonomic activity test, AMPSc enhanced the horizontal movements of AD mice relative to controls (*P* < 0.05; Figure 4(a)), but failed to influence vertical movements (*P* > 0.05; Figure 4(b)). In a fatigue rotarod test, AMPSc enhanced the endurance times of AD mice by >25% (*P* < 0.01; Figure 4(c)) but had no significant effects on control mice (Figure 4(c)).

The water maze test is commonly used to evaluate learning and memory in animals [16]. Here, we applied this test to evaluate the effects of AMPS on the cognitive abilities of AD mice. We initially observed a >15% enhancement in the escape latency times of AD mice (*P* < 0.01; Figure 4(d)). A 4-week course of AMPSc administration led to a nearly 20% decrease in the escape latency times (*P* < 0.05; Figure 4(d)). AMPSc failed to influence the escape latency times of control mice (*P* > 0.05; Figure 4(d)).

TUNEL staining was used to analyze the apoptotic statuses of hippocampal neurons. In both control and AMPSc-treated mice, we observed few TUNEL-positive cells, suggesting that a minority of neurons were apoptotic. Larger amounts of TUNEL-positive apoptotic neurons were noted in AD mice, whereas a 4-week course of AMPSc administration strongly reduced apoptosis in this population, as demonstrated by the reduction in green fluorescence intensity (Figure 4(e)).

### 3.4. AMPSc Regulated Ach, AchE, and ChAT Concentrations in Serum and Hypothalamus

**Figure 5:**
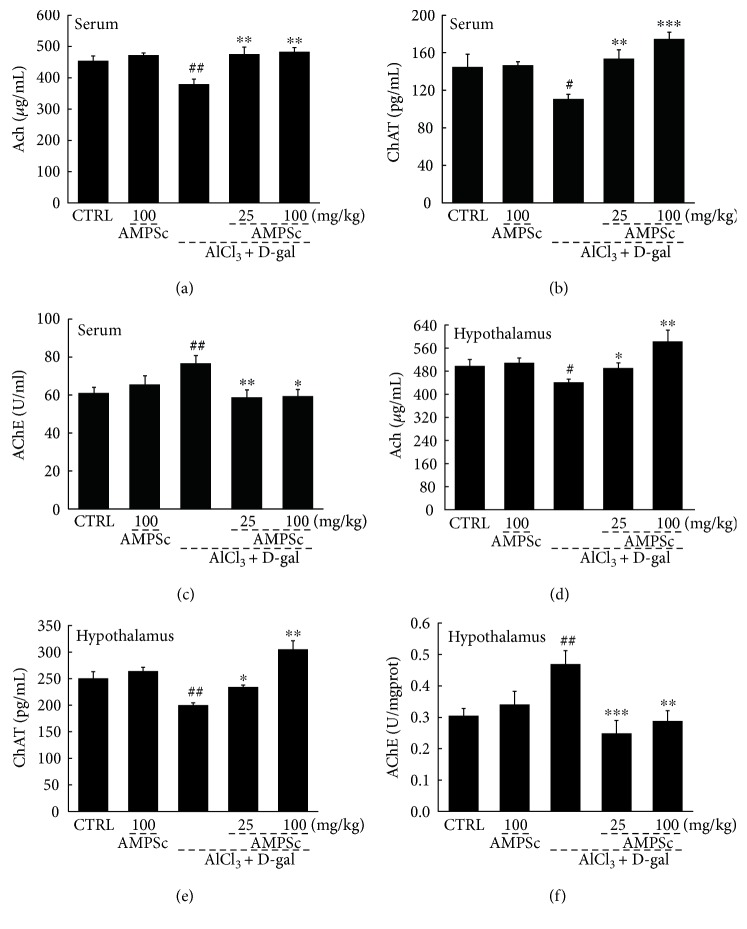
AMPSc enhanced the levels of (a and d) Ach and (b and e) ChAT and reduced the levels of (c and f) AchE in serum and hypothalamus of AD mice detecting via ELISA method. Data are expressed as mean ± S.E.M. (*n* = 10). ^#^*P* < 0.05 and ^##^*P* < 0.01 versus normal mice (CTRL); ^∗^*P* < 0.05, ^∗∗^*P* < 0.01, and ^∗∗∗^*P* < 0.001 versus AD mice.

We noted significant reductions in the serum and hypothalamic Ach and ChAT concentrations, which were accompanied by increased AchE concentrations, in AlCl_3_ and D-gal-induced AD mice relative to control mice (*P* < 0.05; Figure 5()), suggesting disruption of the central cholinergic function. Compared to AD mice, AMPSc increased both the Ach and ChAT levels and reduced the AchE levels in the serum and hypothalamus in a dose-dependent manner (*P* < 0.05; Figure 5()).

### 3.5. AMPSc Regulated Oxidative Status in the Serum and Hypothalamus

Oxidative stress is the basis for an important hypothesis regarding the pathophysiology of neurodegenerative disorders. Compared with control mice, AMPSc alone significantly enhanced the serum and/or hypothalamic levels of SOD and GSH-Px and reduced the levels of ROS in AD mice (*P* < 0.05; Table 2). Compared with AD mice, a 4-week course of AMPSc administration yielded in >50% and 20% increases in SOD and GSH-Px activities, resp., and a >45% reduction in ROS levels in the serum and/or hypothalamus (*P* < 0.01; Table 2).

### 3.6. AMPSc Regulated A*β* Levels in the Serum and Hippocampus

**Figure 6:**
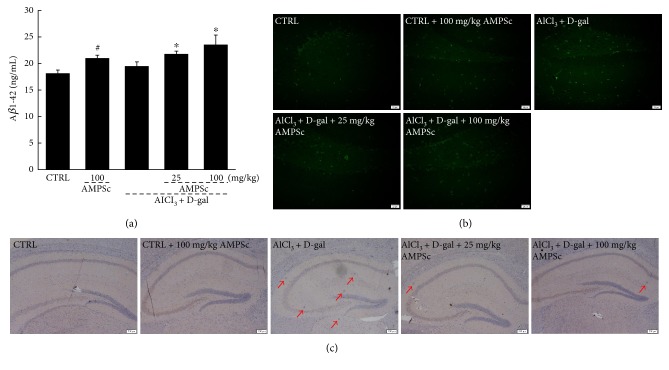
Effect of AMPSc on A*β* clearance in the blood and hippocampus. (a) The levels of A*β* in serum were significantly enhanced by AMPS. Data are expressed as mean ± S.E.M. (*n* = 10). ^#^*P* < 0.05 versus normal mice (CTRL), ^∗^*P* < 0.05 versus AD mice. AMPS significantly reduced A*β* aggregates in hippocampus of AD mice analyzed via (b) thioflavin-S fluorescence staining (*n* = 6; scale bar: 20 *μ*m) and (c) immunohistochemistry staining (*n* = 6; scale bar: 200 *μ*m). AMPS: *A. mellea* polysaccharides.

A*β*, which exhibits strong aggregating properties, is considered the core component of amyloid plaques. Compared with control mice, we observed no significant changes in the serum A*β*1-42 levels in AD mice, whereas a 4-week course of AMPSc led to a >20% increase in serum A*β*1-42 concentrations (*P* < 0.05; Figure 6(a)). Furthermore, AMPSc also increased the serum A*β*1-42 levels in control mice (*P* < 0.05; Figure 6(a)). In the hippocampus, AMPSc suppressed the strong expression of A*β* in AD mice, as indicated by the reduction in green fluorescence intensity (Figure 6(b)). The suppressive effects of AMPSc on A*β*1-40 deposition were also confirmed by immunohistochemistry (Figure 6(c)).

### 3.7. AMPSc Regulated Oxidative Damage and p-Tau Aggregations in Hippocampus

**Figure 7:**
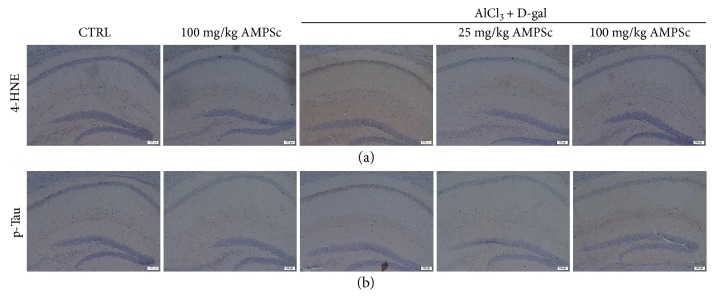
The effects of AMPSc on (a) 4-HNE expression levels and (b) p-Tau aggregations in hippocampus of AD mouse via immunohistochemistry staining (*n* = 6) (scale bar: 100 *μ*m). AMPS: *A. mellea* polysaccharides.

Compared to control mice, high expression levels of 4-NHE (Figure 7, a) and excessive aggregations of p-Tau (Figure 7, b) in the hippocampus were noted in AD mice. In contrast, four-week AMPSc treatment strongly reduced the expression levels of 4-NHE (Figure 7, a) and attenuated the aggregations of p-Tau in AD mice (Figure 7, b).

## 4. Discussion

By 2050, the number of patients suffering with dementia is expected to reach 115.4 million [17]. Our present study successfully confirmed the neuroprotective effects of AMPS in L-Glu-induced HT22 apoptotic cells and a chemically induced AD mouse model, as evidenced by the significant amelioration of nuclear and mitochondrial apoptosis. Furthermore, a clinical decline in short-term memory is considered a symptom of AD, and AMPS was shown to affect the behavior of AD mice. In contrast to other agents used to treat AD, AMPS contains multiple polysaccharides that affect systemic targets and exert various functions, such as antioxidative and antiapoptotic effects, to eliminate the symptoms of AD in a much more natural manner.

In our *in vitro* study, the robust protection provided by AMPS against apoptosis was associated with the inhibition of ROS overproduction and the reversal of MMP depolarization. ROS accumulation causes oxidative stress and thus leads to cellular dysfunction and apoptosis [18], which are associated with the opening of the mitochondrial permeability transition pore [19]. Within a feedback loop, MMP dissipation leads to further ROS release from the mitochondria to the cytoplasm [20], while activating other proapoptotic molecules such as caspase-3 [21]. Caspase-3 is an active component of proteolytic cleavage, which directs the execution of the apoptotic program [22]. Our data obtained from L-Glu-induced HT22 apoptotic cells suggest an association between AMPS-mediated neuroprotection and oxidative stress-mediated mitochondrial apoptotic signaling.

In the present study, our AlCl_3_ and D-gal-induced AD mice exhibited signs of enhanced oxidative stress. As a biomarker of oxidative damage, 4-HNE is a cytotoxic end product of lipid peroxidation, which is essential for cell survival signaling [23]. The increase of 4-HNE triggers inflammatory responses and elevates ROS [24]. Comparatively, AMPS induced significant antioxidative effects, as shown by the suppression on 4-HNE expressions, the reductions in ROS levels, and increases in the activities of the endogenous antioxidants SOD and GSH-Px, which play an important role in removing oxygen-free radicals. AlCl_3_ has been reported to induce the generation of free radicals and neurotoxicity in the brain, which might lead to degenerative disorders [25]. Over the long term, D-gal injections not only induce impairments in learning and memory but also cause mitochondrial dysfunction and ROS accumulation in the brain [26]. The brain contains large amounts of polyunsaturated fatty acids, and its structure, which can be damaged by oxidation of proteins and lipids, is very sensitive to oxidative stress [27]. In AD, oxidative stress damage causes neuronal cell apoptosis by destroying the balance between ROS generation and mitochondrial removal [28]. D-gal induced the dissipation of MMP, and neurodegeneration is promoted by caspase-mediated apoptosis, which mainly occurs in the dentate gyrus (DG) region of the hippocampus [29]. Using TUNEL staining, we confirmed that AMPS successfully suppressed neuronal apoptosis in the hippocampus, compared to nontreated AD mice. Together with our *in vitro* data, these results demonstrate that the AMPS-induced improvements in the cognitive performances of AD mice may be related to its antioxidant activities, which led to further suppression of apoptosis.

AMPS also enhanced the serum levels of A*β* while reducing the hippocampal expression of A*β*. The overproduction of A*β* protein and resulting formation of intracellular neurofibrillary tangles lead to the generation of extracellular senile plaques, which serve as the pathological index in the brain of a rodent with AD [30]. A*β* aggregation induces oxidative stress and mitochondrial dysfunction and leads to the production of ROS, which are involved in the pathogenesis of AD [31]. In a normal physiological state, A*β* can be detected in the blood and cerebrospinal fluid as it is slowly removed from the brain into the periphery via the transport mechanism and enzyme degradation. In AD patients, the clearance of A*β* accumulated in the brain may cause the increased levels of A*β* in the peripheral blood [32]. As reported, the fruit of *Cornus officinalis*, a traditional medicinal agent, exerts neuroprotective activity and significantly increases the plasma levels of A*β* [33]. On the other hand, the deposits of A*β* trigger the deficits of memory and synaptic degeneration, which further result in the neuronal signaling downstream of p-Tau pathology. The deposition of tau protein due to abnormal phosphorylation and glycosylation modification eventually leads to the formation of neurofibrillary tangles, which is related to the existence of excessive A*β* and plaques, proving the tau pathology in AD. We found that the ability of AMPS to reduce the hippocampal deposition of A*β* in mice played a central role in its ability to improve AD-like behaviors in mice.

The cholinergic system, which involves neurotransmitters such as Ach, is essential for the establishment, storage, and recovery of long-term memory. As reported, the decreases in Ach and ChAT release and enhancement of AchE activity caused by an impaired cholinergic system are key alterations affecting the cognitive deficit characteristic of AD pathogenesis [34]. Ach, ChAT, and AchE are among the neurotransmitters with crucial roles in synaptic transmission, which is related to memory and learning deficits [35]. *H. erinaceus* extracts were previously found to improve the AlCl_3_ and D-gal-induced impairment of learning and memory in mice by regulating Ach and ChAT levels [9]. Similarly, the modulatory effects of AMPS on neurotransmitters might define an important protective role of cholinergic function in AD mice.

Our present study had some limitations. First, although we isolated polysaccharides from *A. mellea* mycelia, we could not obtain sufficient purity for a structural analysis. Further investigation is required. Second, the relationships among oxidative stress, neurotransmitter levels, and A*β* deposition should be investigated in greater detail.

In conclusion, our results demonstrate that AMPS protects against L-Glu-induced neurotoxicity in HT22 cells and mitigates AD-like behaviors in an AlCl_3_ and D-gal-induced mouse model of AD. These effects might be largely attributable to the ability of AMPS to modulate oxidative stress. Our findings provide experimental evidence that *A. mellea* might be a useful neuroprotective agent for the treatment or prevention of neurodegenerative disease.

## Figures and Tables

**Table 1:** Effect of different ratios of ethanol on the polysaccharides yield from *A. mellea* mycelium.

Ethanol concentration	Name	Yield (%)
50%	AMPSa	0.93
60%	AMPSb	1.30
70%	AMPSc	1.93
80%	AMPSd	1.60
90%	AMPSe	1.00

**Table 2:** The effects of AMPSc on oxidative statues in serum or hypothalamus in AD mice.

		CTRL	CTRL + AMPSc (mg/kg)	AlCl_3_ + D-gal	AlCl_3_ + D-gal + AMPSc (mg/kg)	
			100		25	100
Serum	SOD (U/mL)	98.6 ± 7.4	117.0 ± 5.7^#^	75.9 ± 3.9^#^	110.3 ± 5.3^∗∗^	111.5 ± 9.0^∗∗^
	GSH-Px (U/mL)	249.8 ± 13.1	251.3 ± 5.7	213.4 ± 10.5^##^	254.4 ± 9.0^∗∗^	290.9 ± 16.0^∗∗∗^
Hypothalamus	SOD (U/mgprot)	44.4 ± 2.1	55.9 ± 4.6^#^	30.1 ± 2.6^##^	47.0 ± 4.8^∗∗^	66.1 ± 6.4^∗∗∗^
	GSH-Px (U/mL)	326.1 ± 13.9	385.0 ± 27.2^#^	282.3 ± 16.4^#^	405.7 ± 31.1^∗∗^	501.4 ± 15.2^∗∗∗^
	ROS (FI/mgprot)	23087.4 ± 1905.5	15564.8 ± 3030.9^#^	34418.8 ± 3986.2^#^	14104.1 ± 1260.5^∗∗^	17877.6 ± 2713.4^∗∗∗^

Treatment with AMPSc and the levels of SOD, GSH-Px, and ROS in serum and/or hypothalamus were detected via ELISA method. Data are expressed as mean ± S.E.M. (*n* = 10). ^#^*P* < 0.05 and ^##^*P* < 0.01 versus normal mice (CTRL); ^∗∗^*P* < 0.01 and ^∗∗∗^*P* < 0.001 versus AD mice.

## Abbreviations

ANOVA: One-way analysis of variance
Ach: Acetylcholine
AchE: Acetylcholine esterase
AD: Alzheimer's disease
AMPS: *A*. *mellea* mycelium polysaccharides
A*β*: Amyloid beta
ChAT: Choline acetyltransferase
DG: Dentate gyrus
D-gal: D-Galactose
ELISA: Enzyme-linked immunosorbent assay
GSH-Px: Glutathione peroxidase
L-Glu: L-Glutamic acid
MMP: Mitochondrial membrane potential
ROS: Reactive oxygen species
SOD: Superoxide dismutase
4-HNE: 4-Hydroxynonenal.
